# Diagnostic value of deep learning-assisted endoscopic ultrasound for pancreatic tumors: a systematic review and meta-analysis

**DOI:** 10.3389/fonc.2023.1191008

**Published:** 2023-07-27

**Authors:** Bing Lv, Kunhong Wang, Ning Wei, Feng Yu, Tao Tao, Yanting Shi

**Affiliations:** ^1^ School of Computer Science and Technology, Shandong University of Technology, Zibo, Shandong, China; ^2^ Department of Gastroenterology, Zibo Central Hospital, Zibo, Shandong, China

**Keywords:** pancreatic tumor, artificial intelligence, deep learning, endoscopic ultrasound, meta-analysis, systemic review

## Abstract

**Background and aims:**

Endoscopic ultrasonography (EUS) is commonly utilized in the diagnosis of pancreatic tumors, although as this modality relies primarily on the practitioner’s visual judgment, it is prone to result in a missed diagnosis or misdiagnosis due to inexperience, fatigue, or distraction. Deep learning (DL) techniques, which can be used to automatically extract detailed imaging features from images, have been increasingly beneficial in the field of medical image-based assisted diagnosis. The present systematic review included a meta-analysis aimed at evaluating the accuracy of DL-assisted EUS for the diagnosis of pancreatic tumors diagnosis.

**Methods:**

We performed a comprehensive search for all studies relevant to EUS and DL in the following four databases, from their inception through February 2023: PubMed, Embase, Web of Science, and the Cochrane Library. Target studies were strictly screened based on specific inclusion and exclusion criteria, after which we performed a meta-analysis using Stata 16.0 to assess the diagnostic ability of DL and compare it with that of EUS practitioners. Any sources of heterogeneity were explored using subgroup and meta-regression analyses.

**Results:**

A total of 10 studies, involving 3,529 patients and 34,773 training images, were included in the present meta-analysis. The pooled sensitivity was 93% (95% confidence interval [CI], 87–96%), the pooled specificity was 95% (95% CI, 89–98%), and the area under the summary receiver operating characteristic curve (AUC) was 0.98 (95% CI, 0.96–0.99).

**Conclusion:**

DL-assisted EUS has a high accuracy and clinical applicability for diagnosing pancreatic tumors.

**Systematic review registration:**

https://www.crd.york.ac.uk/prospero/display_record.php?ID=CRD42023391853, identifier CRD42023391853.

## 1 Introduction

Pancreatic tumors (PTs) are relatively common tumors of the digestive tract. Benign PTs include serous cystadenomas, mucinous cystadenomas, and intraductal papillary mucinous neoplasms (IPMNs), while malignant tumors include pancreatic ductal adenocarcinomas (PDACs), pancreatic neuroendocrine tumors (PNETs), and pancreatic adenosquamous carcinomas (PASCs). Overall, PDAC, which has a high degree of malignancy, is the most common type of pancreatic cancer (PC), and owing to a lack of obvious symptoms in the early stages along with rapid progression, it is often detected at a late stage (1). Studies have shown that the five-year survival rate for PDAC is only 8–10% (2). Different degrees of malignancy in PT, however, result in significantly different prognoses. PNET, for example, has a 5-year survival rate of > 60% when diagnosed as pathological grade 1 or 2, which are low-grade malignancies, whereas those diagnosed as grade 3, or a high-grade malignancy, have a 5-year survival rate of < 30% (3–5). The accurate and timely identification and staging of PT can help determine patient prognosis and the appropriate course of treatment.

Currently, computed tomography (CT), magnetic resonance imaging (MRI), and endoscopic ultrasound (EUS) are the primary modalities utilized for the diagnosis of PT. MRI and CT, however, are less sensitive for monitoring smaller pancreatic lesions, and also for differentiating between benign and malignant tumors (6, 7). By combining endoscopy with ultrasound, EUS provides a more accurate and complete display of the pancreatic structure and visualization of space-occupying lesions (8), and previous studies have shown that EUS performs well in the diagnosis of a variety of pancreatic masses, with higher accuracy than many other clinical diagnostic techniques (9, 10). Additionally, EUS-guided fine-needle aspiration/biopsy (EUS-FNA/EUS-FNB) allows for the quick and easy sampling of pathological tissue, further improving the accuracy of PT diagnoses (11). The primary method for the imaging-based diagnosis of PT in clinical practice still relies heavily on the visual judgment of the individual operating the endoscope, which is overly dependent on their experience, and can lead to missed diagnoses or misdiagnosed cases as the result of insufficient experience, fatigue, or distraction. Computer-aided diagnosis/detection (CAD) analyses medical image data and other data using computer technology to assist practitioners in more objectively, quickly, and accurately completing diagnostic work. Many studies have verified the feasibility of utilizing CAD in the process of image-based diagnosis (12–14).

In recent years, artificial intelligence (AI) technology has been increasingly utilized in various fields of medicine, such as image analysis, diagnostic recommendations, and clinical risk prediction, which has reduced medical errors, to a certain extent, and improved diagnostic efficiency (15). Sunwoo et al. (16), for example, used AI technology to analyze the diagnosis of brain metastases from MRI scans, and the sensitivity increased from 77.6% to 81.9%, while the reading time decreased from 114.4 seconds to 72.1 seconds. There are two primary methods for utilizing AI in the analysis of medical images for assisted diagnosis: diagnosis based on traditional machine learning methods and diagnosis based on deep learning (DL) methods.

As a branch of AI, traditional machine learning-based methods primarily involve the manual extraction of features and the selection of suitable classifiers for statistical analysis. DL, in turn, is a subset of machine learning. At the 2012 ImageNet Large Scale Visual Recognition Challenge (17), Krizhevsky et al. (18) proposed AlexNet, a deep convolutional neural network, that overwhelmingly won the competition and triggered a wave of DL in various fields. Compared to traditional machine learning, DL automates feature extraction in a data-driven manner, and is capable of learning deeper and more abstract features from the target data (19, 20). DL significantly improves accuracy in areas such as image classification, object detection, and semantic segmentation, and its performance exceeds that of traditional machine learning techniques (19, 21).

A previous meta-analysis showed that practitioners using EUS for the diagnosis of PT had a sensitivity of 85% (95% confidence interval [CI], 69–94%), specificity of 58% (95% CI, 40–74%), and accuracy of 75% (95% CI, 67–82%) (6). Dumitrescu et al. (22) conducted a meta-analysis of AI-assisted EUS for PC diagnosis, which included 10 studies; three used traditional machine learning techniques, and seven used DL techniques. The pooled sensitivity for the AI diagnoses was 92% (95% CI, 89–95%), and the pooled specificity was 90% (95% CI, 83–94%). We are hopeful that the results of these studies can be compared with the results of our meta-analysis as a way to evaluate the advantages of DL-assisted EUS for the diagnosis of PC.

In the present study, the accuracy of DL-assisted EUS in the diagnosis of PT was quantified through a meta-analysis, which aimed to provide comprehensive and objective evidence for its utilization in clinical practice. The primary outcome of the present study was the overall performance of DL in diagnosing PT, while the secondary outcome was the ability to compare DL and practitioners performing traditional EUS.

## 2 Methods

The present study followed the Preferred Reporting Items for Systematic Review and Meta-Analysis of Diagnostic Test Accuracy Studies (PRISMA-DTA) guidelines (23), the checklist for which is presented in **Supplementary Table S1**. Prior to its onset, the present study was registered with the International Prospective Register of Systematic Reviews (PROSPERO) (24) on January 25, 2023 (ID: CRD42023391853), and because all of the data analyzed were collected from the included literature, ethical approval was not required.

### 2.1 Search strategy

We performed searches for the present meta-analysis in four commonly used databases: PubMed, Embase, Web of Science, and the Cochrane Library database. The final search was conducted on February 21, 2023, and included all articles from the four databases, beginning at the time of their creation and ending at the time of the final search. The keywords which were searched relating to DL included “deep learning”, “artificial intelligence”, “machine learning”, “computer-aided”, “natural networks”, “image classification”, “object detection”, and “semantic segmentation”; those relating to EUS included “ultrasonography”, “ultrasound”, and “EUS”; and those relating to PT included “pancreas” and “pancreatic”. The detailed search strategy is presented in **Supplementary Table S2**.

### 2.2 Study selection

The inclusion criteria for the present study were as follows (1): studies using DL to detect PT; (2) detection based on EUS images or videos; (3) use of pathological findings or expert labeling as diagnostic criteria; (4) detailed description of the source and composition of the training and test sets; and (5) true positive (TP), false positive (FP), true negative (TN), and false negative (FN) values were obtained directly or indirectly. For studies with missing data, the corresponding author was contacted via email in order to fill in the blanks.

The exclusion criteria were as follows: (1) articles without raw data, such as reviews, comments, or letters; (2) not full-text articles; (3) TP, FP, TN, and FN data not included, or no response received from the corresponding author via email when attempting to gather the missing data.

The initial articles returned from the searches were screened for inclusion by KW and NW, based on the aforementioned criteria, and any disagreements were resolved through discussions with BL.

### 2.3 Data extraction

KW and TT independently extracted data from the included studies, and resolved any disagreements through discussion. The following information was collected from each included study: first author, year of publication, country or region, diagnostic criteria, number of patients, data source, number of training sets, DL algorithms, sensitivity, and specificity. For studies with multiple test results, we extracted the resulting data in the following order: prospective test set, external test set, and test set with the largest sample size. We also extracted diagnostic data regarding the EUS practitioners for comparison with the DL models.

### 2.4 Quality assessment

We utilized the Quality Assessment of Diagnostic Accuracy Studies version 2 (QUADAS-2) to assess the quality of the included studies, although to more accurately assess the DL models, we supplemented the patient selection section with the following questions: (1) “Was the composition of the training and test sets described?”; and (2) “Were imaging modalities and image/video quality described in detail?”. We also added the following questions to the index test section: (1) “Was the algorithm development and training processes described?”; and (2) “Does the model be evaluated using an independent test set?”.

### 2.5 Statistical analysis

We conducted our meta-analysis using a bivariate random-effects model to evaluate the performance of DL in the diagnosis of PT. We plotted a summary receiver operating characteristic (SROC) curve, and calculated the pooled sensitivity, specificity, positive likelihood ratio (PLR), negative likelihood ratio (NLR), diagnostic odds ratio (DOR), area under the SROC curve (AUC), and 95% CIs. High sensitivity and PLR indicated that the DL model was suitable for confirming the diagnosis of PT; high specificity and low NLR indicated that the DL model was good at excluding patients who did not have the disease; and DOR and AUC are overall measures of diagnostic accuracy, with a high DOR and AUC indicating that the DL model was good at confirming and excluding PT.

Statistical heterogeneity was determined by the I^2^ statistic as follows: < 30% indicated low heterogeneity; 30–60% indicated moderate heterogeneity; and > 60% indicated high heterogeneity. Publication bias was analyzed using Deeks’ funnel plot asymmetry test, for which *P* < 0.05 indicated publication bias. We utilized subgroup analysis and meta-regression to identify sources of heterogeneity, and also to explore the diagnostic performance of the different subgroups, and we used Fagan plots to assess the clinical applicability of DL for the diagnosis of PT.

The quality of the included studies was assessed using Review Manager 5.4 (Cochrane Collaboration, Oxford, UK), while other statistics and charts were obtained using Stata/SE 16.0 (Stata, College Station, TX, USA).

## 3 Results

### 3.1 Included studies and quality assessment

**Figure 1:**
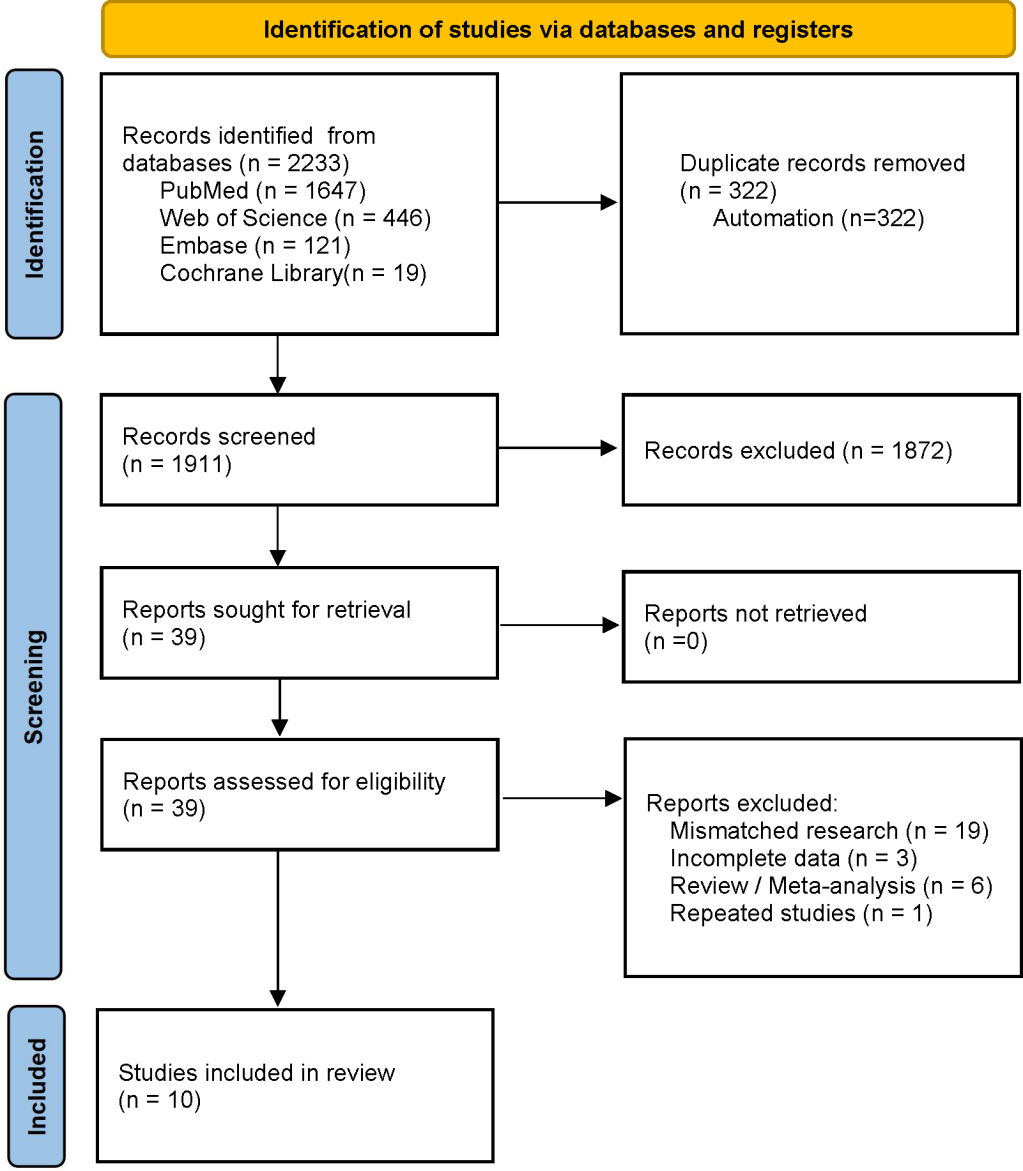
Preferred Reporting Items for Systematic Review and Meta-Analysis of Diagnostic Test Accuracy Studies (PRISMA) flow diagram for study selection.

Our initial search yielded 2,233 relevant articles, of which 322 duplicates were automatically removed by the software and 1,872 that were not relevant were manually excluded after reading the titles and abstracts. After reading the full-text, a total of ten articles were included in the present meta-analysis (25–34). The data extraction process is shown in **Figure 1**, and the details of the included studies are listed in **Table 1**.

**Table 1:** Details of the included studies.

Study	Country/ Region	Study Center	Study design	Imaging type	Algorithm	Standard Reference	Patients (n)	Train set images(n)	Test set	Tester	Sensitivity	Specificity
Tonozuka 2021 (25)	Japan	Single	Retrospective	EUS	Customized CNN	Pathology	139	920	Internal Image	DL	0.924	0.841
Udriștoiu 2021 (26)	Romania	Single	Retrospective	Gray-Scale CHI CDI RTE	Customized CNN	Pathology	65	2688	Internal Image	DL	0.9821	0.9955
Oh 2021 (27)	Korea	Multi	Retrospective	EUS	Attention U-Net	Pathology	111	43	External Image	DL	0.723	0.989
Huang 2022 (28)	China	Single	Retrospective	CEUS	SE-ResNeXt	Pathology	104	2480	Internal Patient	DL	0.75	0.83
Kuwahara 2022 (29)	Japan	Single	Retrospective	EUS	EfficientNetV2	Pathology	933	18318	Internal Video	DL	0.94	0.82
Tian 2022 (30)	China	Single	Retrospective	EUS	YoloV5	Pathology	157	807	Internal Patient	DL	0.95	0.75
										practitioner	0.8	0.875
Tong 2022 (31)	China	Multi	Retrospective	CEUS	ResNet	Pathology	558	351	External Image	DL	0.922	0.8571
										practitioner	0.857	0.81
VilasBoas 2022 (32)	Portugal	Single	Retrospective	EUS	Xception	Pathology	28	4404	Internal Image	DL	0.983	0.989
Seo 2022 (33)	Korea	Single	Retrospective	EUS	DAF-Net	Pathology	150	330	Internal Image	DL	0.84	0.981
Tang 2023 (34)	China	Multi	Prospective	CEUS	UNet++	Pathology	1,284	4432	Internal Video	DL	0.923	0.923
										practitioner	0.885	0.846

CDI, High-MI color Doppler; CEUS, Contrast-enhanced endoscopic ultrasound; CHI, Low-MI contrast-enhancement; MI, Mechanical index; RTE, Real-time elastography.

**Figure 2:**
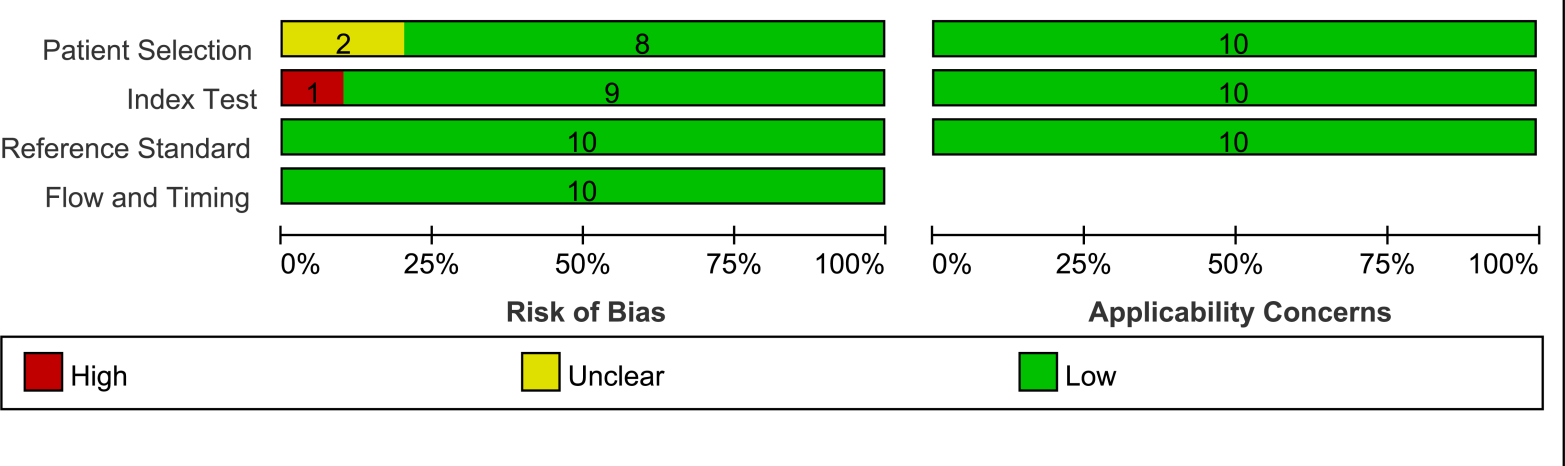
Summary of risk of bias and applicability of concerns graph.

The QUADAS-2 tool was used to assess the quality of the included studies, one of which (26) used data-enhanced images for testing, and was deemed to have a high risk of bias in the index test section, while two (26, 27) failed to describe their patient selection processes and were considered, therefore, to have an unknown risk of bias in the patient selection section. The overall assessment results are shown in **Figure 2**.

The 10 included studies encompassed 3,529 patients, with nine of the studies being retrospective while one was prospective (34). All of the studies used pathological findings as the diagnostic criteria, and seven studies were single-center (25, 26, 28–30, 32, 33) while three were multicenter (27, 31, 34); eight were from East Asia (25, 27–31, 33, 34) and two were from Europe (26, 32); six used plain EUS images (25, 27, 29, 30, 32, 34) while three used contrast-enhanced EUS (CEUS) images (28, 31, 33) and one used grey-scale, low-mechanical index (MI) contrast enhancement, high-MI color Doppler, and real-time elastography multiple imaging techniques (26); six studies used image classification algorithms (25, 26, 28, 30–32), one (30) used object detection algorithms, and three (27, 33, 34) used semantic segmentation algorithms; and six studies (25–27, 31–33) tested the model on an image basis, while four (28–30, 34) tested the model on a patient or video basis. The study aims, participant characteristics, types of lesions, and funding sources of the included studies are listed in **Supplementary Table S3**.

### 3.2 Study characteristics and data extraction

Tonozuka et al. (25) constructed a DL model using convolutional neural networks to identify patients with a normal pancreas (NP) versus those with chronic pancreatitis (CP) and PDAC. A total of 139 patients were included in their study – 76 with PDAC, 34 with CP, and 29 with NP, for whom the sensitivity and specificity were 92.4% and 84.1%, respectively.

Udriștoiu et al. (26) developed a convolutional neural network-based CAD system with long short-term memory neural networks to identify cases of chronic pseudotumoral pancreatitis (CPP), PNET, and PDAC. A total of 65 patients were included in their study – 30 with PDAC, 20 with CPP, and 15 with PNETs. The overall accuracy of their model was 98.26%. In the meta-analysis, we combined the sensitivity and specificity of these models for the diagnosis of PNET and PDAC.

Oh et al. (27) used DL techniques to automatically segment PT on EUS, and their study included 111 patients from 2 hospitals. Their model was tested using internal and external test sets, and the test results were extracted from the external test set for inclusion in the present meta-analysis.

Huang et al. (28) combined DL with traditional machine learning techniques to predict the preoperative invasiveness of PNETs. A total of 104 patients were included in their study, and the AUC of the DL model was 0.81 (95% CI, 0.62–1.00). We only extracted the test results from the DL model for the present meta-analysis.

Kuwahara et al. (29) created a DL model to distinguish between pancreatic and non-pancreatic cancer (NPC) cases, and their study included 933 patients with 9 pancreatic masses, including PDACs, PNETs, and CP. The test results were extracted from the video test set, and the accuracy and AUC of the DL model were 91% (95% CI, 85–95%) and 0.90 (95% CI, 0.84–0.97), respectively.

Tian et al. (30) performed a real-time diagnosis of PC or NPC based on an object detection algorithm compared with the results of EUS practitioners. Their study included 157 patients, 102 with PC and 55 with NPC. The sensitivity and specificity of their model were 95% and 75%, respectively, while those for the EUS practitioners were 80% and 87.5%, respectively.

Tong et al. (31) created a DL model for differentiating between PDAC and CP. In their study, 558 patients were recruited from 3 hospitals, including 414 patients with PADCs and 144 with CP. Data from one hospital were used for model training and internal testing, while those from the other two were used as the two external test cohorts. We combined the test results of the two external test cohorts for the present meta-analysis.

Vilas-Boas et al. (32) constructed a DL model for the identification of mucinous and non-mucinous pancreatic cystic lesions (PCLs), in which they included a total of 28 patients – 17 with mucinous PCLs and 11 with non-mucinous PCLs. The overall accuracy of their model was 98.5%.

Seo et al. (33) proposed a DL method for PC segmentation. A total of 150 patients with PC were included in this study. The sensitivity and specificity of this model were 89.0% and 98.1%, respectively.

Tang et al. (34) developed a DL-based CAD system to distinguish PC from benign pancreatic masses, for which they retrospectively collected the EUS images of 1,245 patients from multiple centers for training and testing, and also recruited 39 patients for prospective testing. The CAD system achieved an accuracy, sensitivity, and specificity of 93.8%, 90.9%, and 100%, respectively.

We performed a meta-analysis of the aforementioned studies, the results of which were the primary outcomes of the present study. Of the 10 studies included in the present meta-analysis, three (30, 31, 34) compared the diagnostic abilities of the DL model with those of the EUS practitioners. We extracted the data from these three groups and performed a comparative analysis, which was the secondary outcome of the present study.

### 3.3 Performance of DL

**Figure 3:**
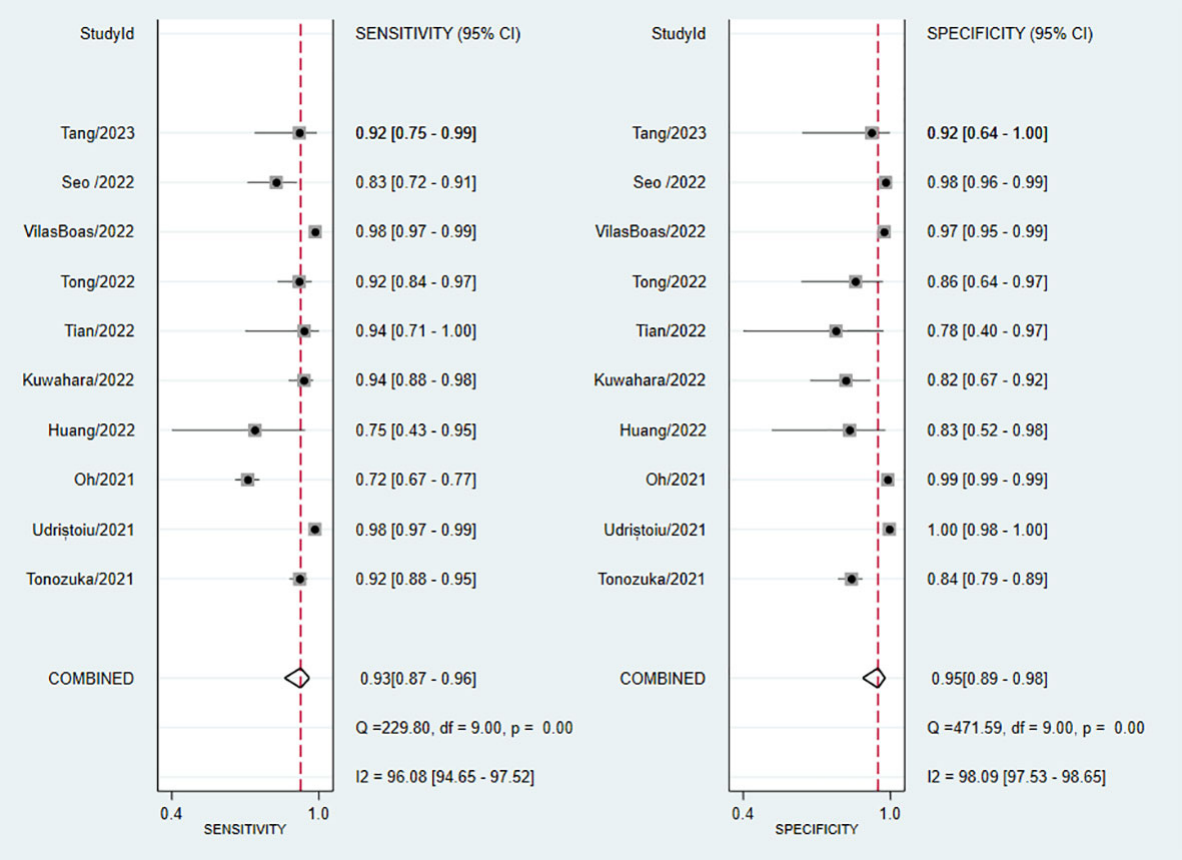
Forest plot of sensitivity and specificity of deep learning (DL) in identifying pancreatic tumors.

**Figure 4:**
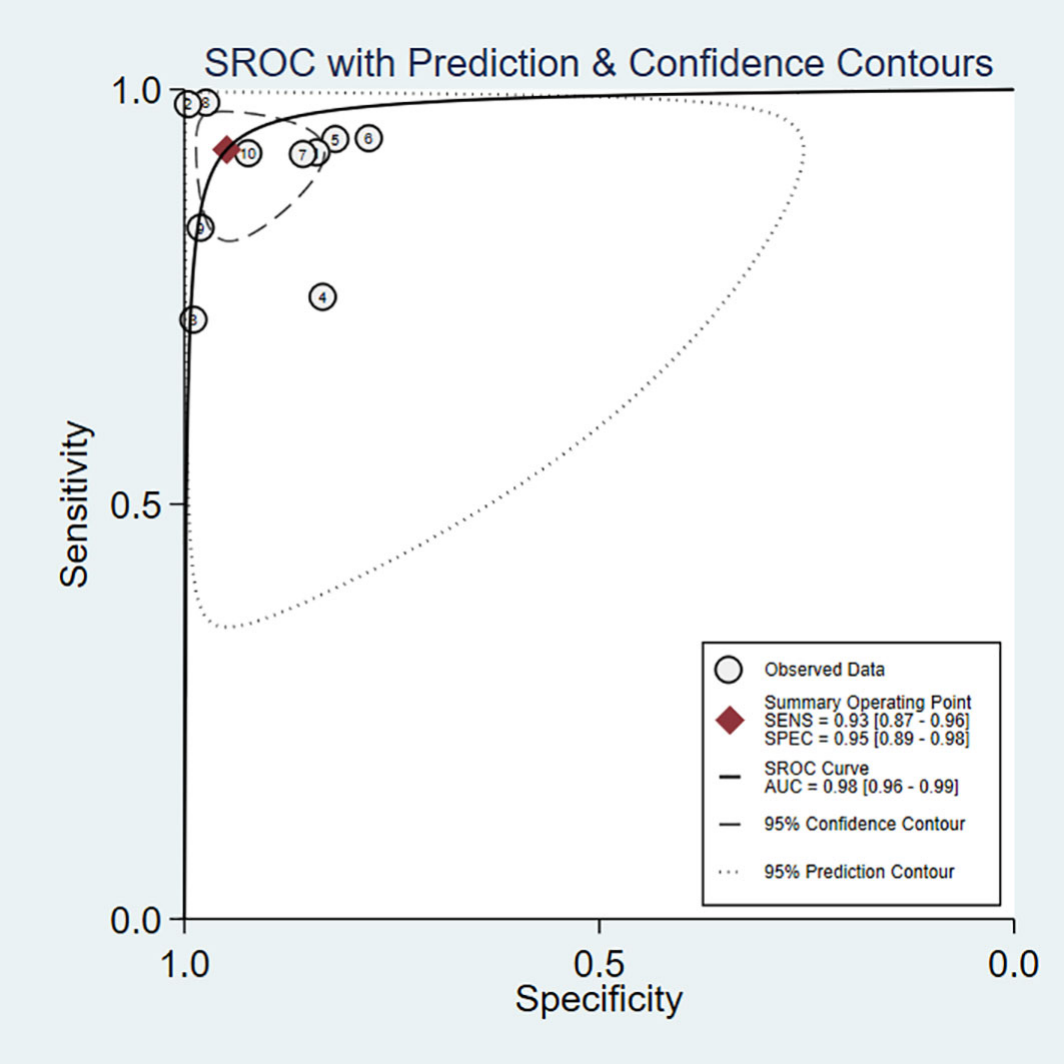
Summary receiver operating characteristic (SROC) curves for the diagnosis of pancreatic tumors using DL. Each circle indicates an individual study, red diamond represents summary sensitivity and specificity.

The pooled sensitivity of DL for diagnosing PT was 93% (95% CI, 87–96%; I^2 = ^96.08%), and the pooled specificity was 95% (95% CI, 89–98%; I^2 = ^98.09%) (**Figure 3**). The PLR was 18.2 (95% CI, 7.91–41.86), the NLR was 0.08 (95% CI, 0.04–0.15), and the DOR was 238.04 (95% CI, 76.3–742.61) (**Supplementary Figures S1, S2**). A PLR > 10 indicates that DL can accurately diagnose PT, while an NLR < 0.1 indicates that DL can effectively exclude PT and a DOR significantly > 1 indicates that DL has good discriminatory ability for PT. We plotted SROC curves to provide a more comprehensive assessment of the performance of the DL model (**Figure 4**), which showed an AUC of 0.98 (95% CI, 0.96–0.99). The AUC value was very close to 1, indicating that DL accurately diagnosed PT.

**Figure 5:**
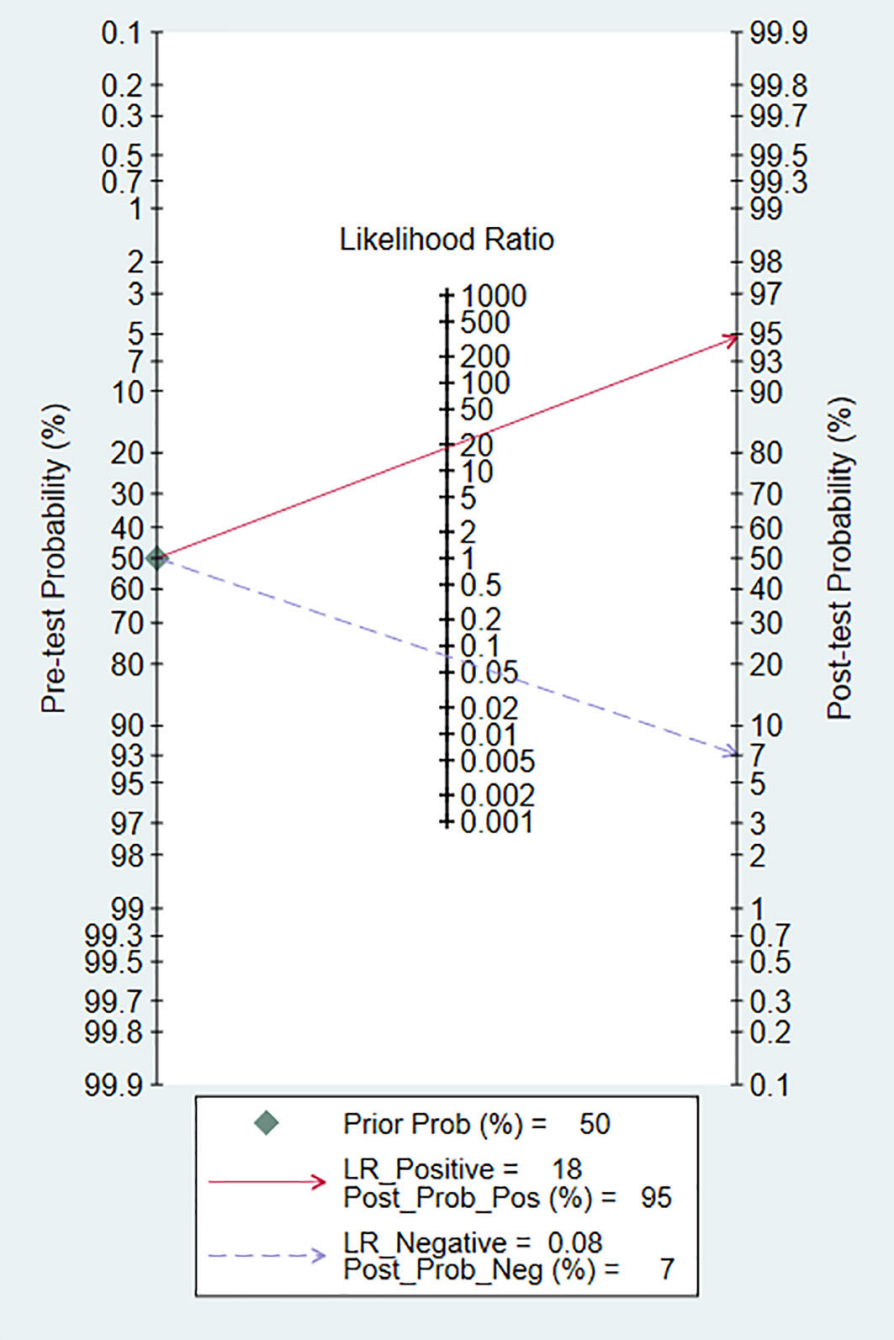
Fagan nomogram of the accuracy of DL in the diagnosis of pancreatic tumors.

We evaluated the clinical application of DL in the diagnosis of PT using Fagan plots (**Figure 5**). When the pre-test probability was set at 50%, the probability of positive patients being diagnosed with PT was 95%, while the probability of negative patients being diagnosed with PT was 7%. These results indicate that DL has a high accuracy, and is an important clinical tool for the diagnosis of PT.

### 3.4 Subgroup analysis and meta-regression

Although the pooled sensitivity, specificity, and DOR showed excellent diagnostic performance for DL, the I^2^ showed high heterogeneity; therefore, we performed a subgroup analysis with meta-regression to analyze the potential sources of heterogeneity. The grouping conditions were as follows: (1) imaging type – normal EUS images vs. other images, such as CEUS; (2) number of training set images – regardless of whether or not the training set had > 1,000 images, using 1,000 divided the 10 studies equally into two parts; (3) test set data type – whether the test data were images, videos, or patients; (4) DL algorithm types – classification vs. other algorithms; and (5) lesion type – solid vs. cystic lesions, the detailed classification is shown in **Supplementary Table S3**. The results of the subgroup analyses showed no statistically significant differences between the subgroups (**Table 2**), indicating that the heterogeneity in the meta-analysis was not due to these factors.

**Table 2:** Subgroup analyses and meta-regression results.

Parameter	Category	Studies(n)	Sensitivity(95%CI)	P	Specificity(95%CI)	P
imaging type	normal EUS	6	0.92(0.86-0.98)	0.18	0.95(0.89-1.00)	0.75
	others	4	0.94(0.87-1.00)		0.95(0.89-1.00)	
training images number	>1000	5	0.96(0.93-0.99)	0.77	0.95(0.90-1.00)	0.90
	<1000	5	0.88(0.80-0.96)		0.94(0.88-1.00)	
test data type	image	6	0.93(0.88-0.98)	0.54	0.97(0.95-0.99)	0.26
	video/patient	4	0.92(0.83-1.00)		0.86(0.71-1.00)	
DL algorithm	classification algorithm	6	0.95(0.92-0.98)	0.94	0.93(0.86-0.99)	0.12
	others	4	0.83(0.71-0.95)		0.98(0.94-1.00)	
lesion type	solid lesions	7	0.93(0.87-0.98)	0.40	0.94(0.88-0.99)	0.25
	contains cystic lesions	3	0.93(0.85-1.00)		0.97(0.93-1.00)	

### 3.5 Sensitivity analysis and publication bias

We further analyzed the sources of heterogeneity in the included studies by performing a sensitivity analysis. After removing each study individually, we examined whether sensitivity, specificity, and the corresponding I^2^ values changed significantly after each change. After removing the study by Oh et al. (27), the sensitivity changed from 93% (95% CI, 87–96%; I^2 = ^96.08%) to 94% (95% CI, 89–97%; I^2 = ^87.1%), with the most significant change in I^2^, although the results still suggested high heterogeneity. Given these results, no source of heterogeneity was identified in the sensitivity analysis, and the overall results of the meta-analysis were considered relatively stable.

**Figure 6:**
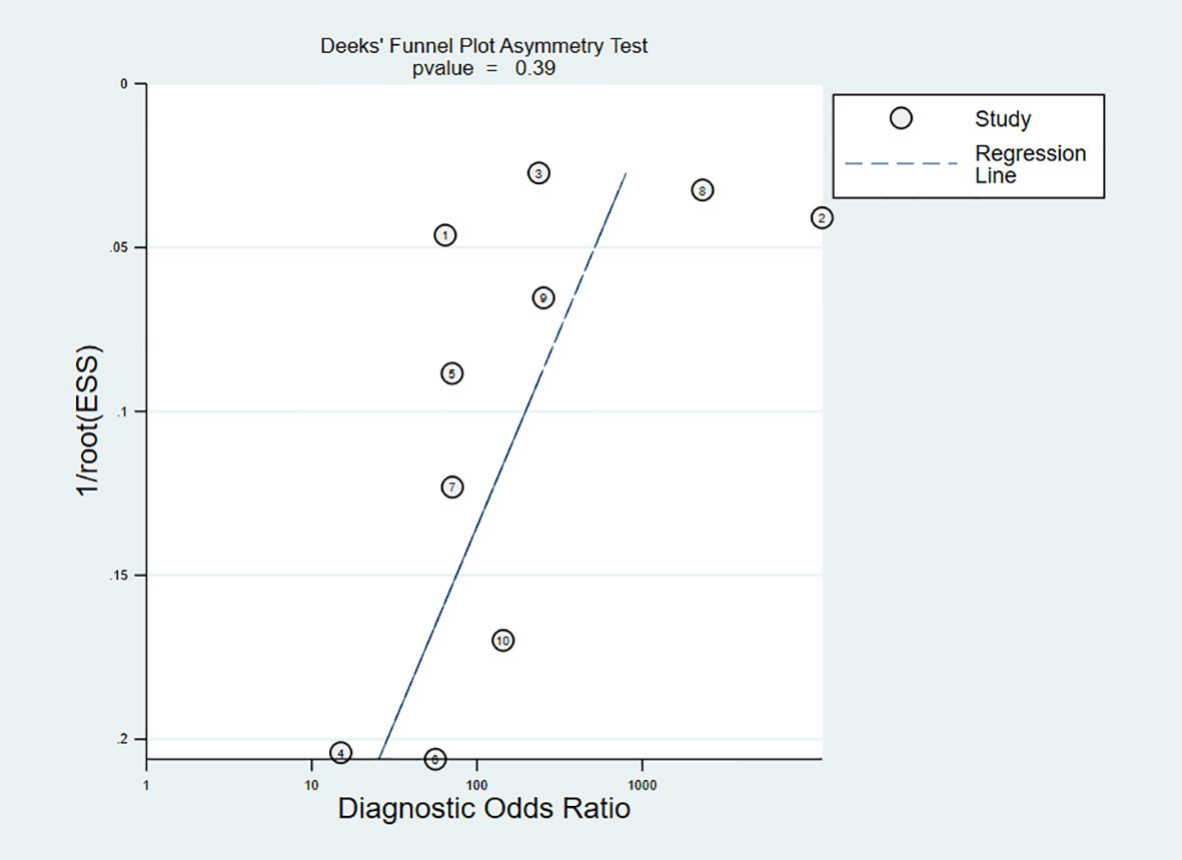
Deeks’ funnel plot asymmetry test for publication.

Publication bias was evaluated using Deeks’ funnel plot (**Figure 6**), which showed *P* = 0.39 (*P >*0.05), indicating that there was no publication bias. Although Deeks’ test was performed, a high publication bias could not definitively be excluded, due to the small number of included studies.

### 3.6 DL vs. EUS practitioners

Of the 10 studies 3 (30, 31, 34) compared DL models with the performance of EUS practitioners (**Table 1**). We performed a subgroup analysis of these three data sets, with a resulting combined sensitivity of 92% (95% CI, 88–97%) vs. 86% (95% CI, 80–92%; *P* = 0.1), and specificity of 86% (95% CI, 76–96%) vs. 84% (95% CI, 73–95%; *P* = 0.37), respectively. Although the DL model performed better than the practitioners, the difference was not statistically significant. As the data from only three groups were included in the comparison, the reliability of the results requires further validation.

## 4 Discussion

DL techniques are being used more and more in clinical practice to significantly improve diagnostic accuracy, stability, and efficiency. In the present study, we performed a meta-analysis to comprehensively evaluate the accuracy of DL-assisted EUS for the diagnosis of PT. A total of 10 studies, encompassing 3,529 patients and 34,773 training images, were included in the present study. The combined sensitivity was 93% (95% CI, 87–96%), specificity was 95% (95% CI, 89–98%), and AUC was 0.98 (95% CI, 0.96–0.99), indicating that the DL-assisted diagnosis of PT is highly accurate. Additionally, we found that the DL model had a better diagnostic ability than that of EUS practitioners, although the difference was not statistically significant.

In the present study, we observed high heterogeneity among the 10 included studies; however, even though subgroup and sensitivity analyses were performed, no sources of heterogeneity were identified. In addition, smaller sample sizes, various DL algorithms, parameter settings, image quality, and EUS devices are possible sources of heterogeneity but need further investigation.

In addition to the high heterogeneity among the included studies, the present meta-analysis had the following limitations (1): most of the included studies were retrospective, while only one was prospective – the clinical applicability of DL, therefore, needs to be validated through more prospective studies; (2) most of the included studies were single-center studies, with only three involving multiple centers – due to differences in equipment and practitioner operating habits, using data from a variety of centers may result in differences in imaging, meaning the generalisability of the single-center trained model requires further validation; (3) most of the included studies involved populations from East Asian, with only two involving European populations, meaning the results of these studies were representative of only a certain population; and (4) some of the included studies involved only a small number of patients, such as one study (30) which included only 28 patients for training and testing, meaning the small sample size may have led to sample bias.

Although we have initially validated the effectiveness of DL models in the diagnosis of PT, these models are still in the clinical exploration stage, and some aspects still need to be improved. One such aspect is the availability of public datasets. Most medical institutions are reluctant to share EUS imaging data for legal purposes, the protection of patient privacy, or for information security, making it difficult for researchers to conduct studies using data from multiple centers. Therefore, there is an urgent need to establish a standard public EUS image database for future research. Another such aspect is open source code. Although most studies used public algorithms, using different parameter settings can affect the results. The availability of open source code, however, could help replicate research and promote the development of this field.

In recent years, emerging EUS-based techniques have shown good performance in the diagnosis of pancreatic lesions (35–37), with one study showing that the accuracy for diagnosing solid pancreatic lesions using wet suction EUS-FNB is 90.4% (35), and a meta-analysis showing that the sensitivity and specificity for detecting malignant pancreatic cystic lesions using EUS-guided through-the-needle biopsy (EUS-TTNB) were 97% and 95%, respectively (36). These techniques, however, require physicians with enhanced expertise and skills to be utilized effectively. As such, one of the included studies constructed a DL-based real-time assisted diagnostic system to guide EUS-FNA and improve the accuracy and efficiency of diagnosing pancreatic masses (34). Combining these new technologies with DL techniques is an important direction for future technological development, and further research is required to improve the efficiency and accuracy of the clinical diagnosis of PT.

The present systematic review provides a comprehensive introduction and quantitative analysis of current research on DL-assisted EUS for the diagnosis of PT. The results of our meta-analysis showed that DL has an excellent diagnostic capability, and can be used as an effective diagnostic aid in clinical practice.

## Data availability statement

The original contributions presented in the study are included in the article/**Supplementary Material**. Further inquiries can be directed to the corresponding author.

## Ethics statement

All of the data for the present study were collected from the referenced literature; therefore, ethical approval was not required.

## Author contributions

YS and BL conceived the idea for the present meta-analysis. BL analyzed the data and wrote the manuscript with the support of the other authors. KW, NW, and TT screened the data. YS and FY provided suggestions for the project and revised the manuscript accordingly. All of the authors discussed the project, and read and approved the final manuscript.
