# Supplementary material for: Diagnostic value of deep learning-assisted endoscopic ultrasound for pancreatic tumors: a systematic review and meta-analysis
Source: Front Oncol. 2023 Jul 27;13:1191008. doi: 10.3389/fonc.2023.1191008 (PMC10414790; doi:10.3389/fonc.2023.1191008)
Supplement: Supplementary file 1 [file DataSheet_1.pdf]

Diagnostic Value of Deep Learning-Assisted Endoscopic Ultrasound for Pancreatic Tumors: A Systematic Review and Meta-Analysis

Bing Lv[1], Ning Wei[2], Feng Yu[2], Tao Tao[2], Yanting Shi[2*]

[1]School of Computer Science and Technology, Shandong University of Technology, Zibo, Shandong, China
[2]Department of Gastroenterology, Zibo Central Hospital, Zibo, Shandong, China
***Correspondence:**
Yanting Shi* yantingshi@hotmail.com

Supplementary File

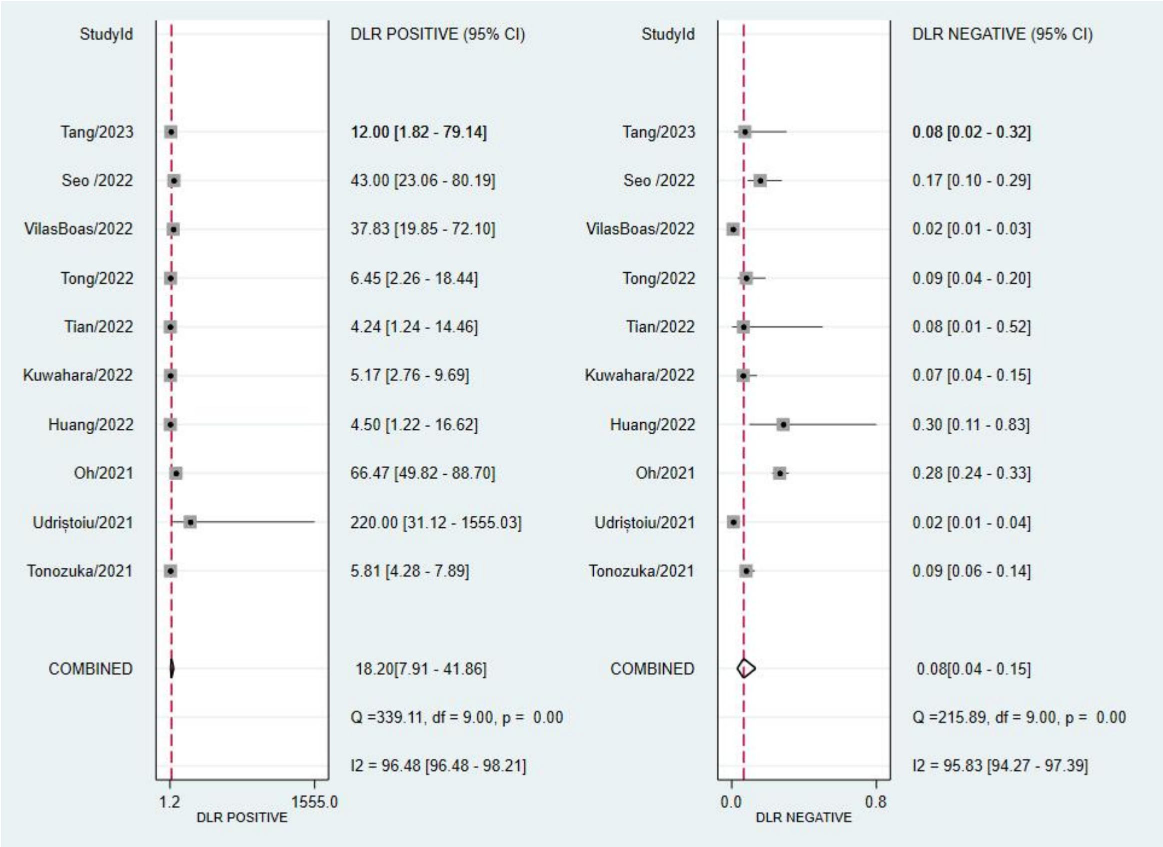

Figure S1. Forest plot of PLR and NLR of DL in identifying pancreatic tumors.

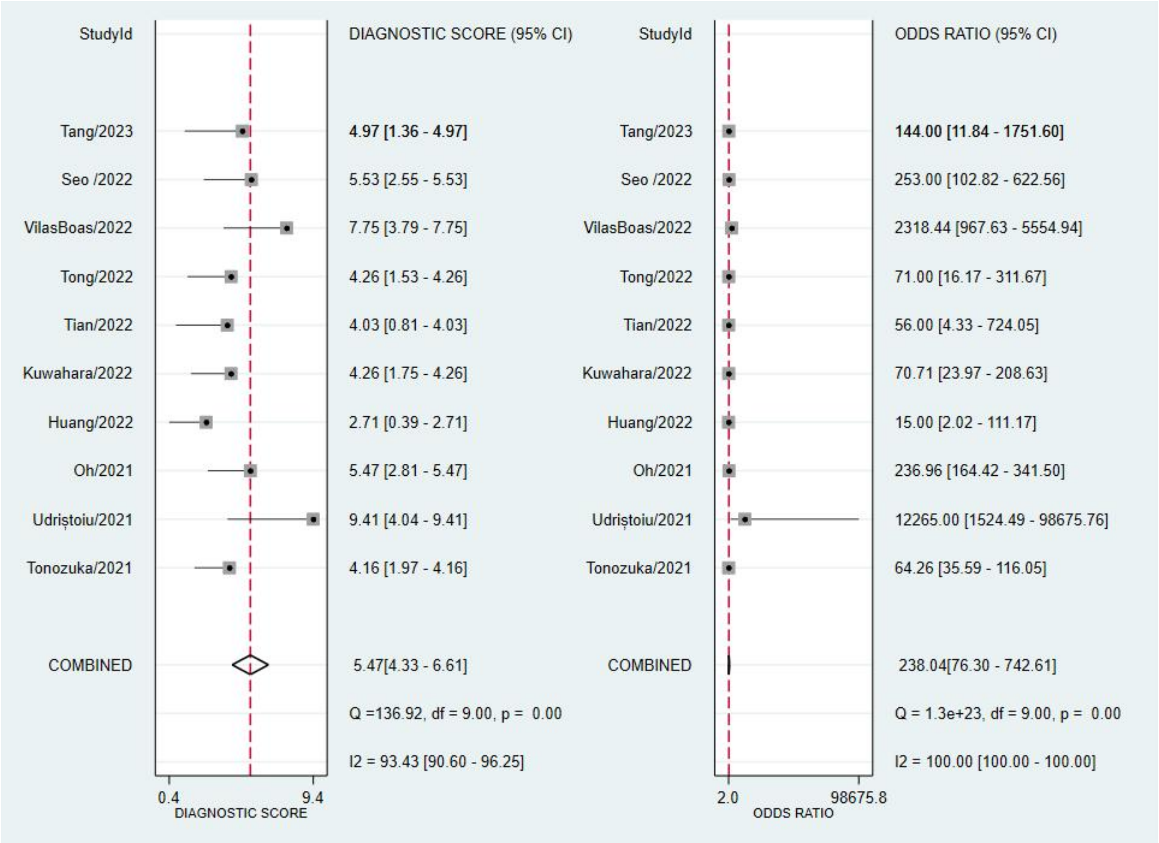

Figure S2. Forest plot for diagnostic odds ratio and diagnostic score after combination.

Table S1. Preferred Reporting Items for Systematic Review and Meta-analysis of Diagnostic Test Accuracy Studies checklist.

Section and Topic	Item #	Checklist item	Reported on page
**TITLE and PURPOSE**			
Title	1	Identify the report as a systematic review (+/- meta-analysis) of diagnostic test accuracy (DTA) studies.	1
Abstract	2	Abstract: See PRISMA-DTA for abstracts.	1
**INTRODUCTION**			
Rationale	3	Describe the rationale for the review in the context of what is already known.	1,2
Clinical role of index test	D1	State the scientific and clinical background, including the intended use and clinical role of the index test, and if applicable, the rationale for minimally acceptable test accuracy (or minimum difference in accuracy for comparative design).	1,2
Objectives	4	Provide an explicit statement of question(s) being addressed in terms of participants, index test(s), and target condition(s).	2,3
**METHODS**			
Protocol and registration	5	Indicate if a review protocol exists, if and where it can be accessed (e.g., Web address), and, if available, provide registration information including registration number.	3
Eligibility criteria	6	Specify study characteristics (participants, setting, index test(s), reference standard(s), target condition(s), and study design) and report characteristics (e.g., years considered, language, publication status) used as criteria for eligibility, giving rationale.	3, Table 1, Table S3
Information sources	7	Describe all information sources (e.g., databases with dates of coverage, contact with study authors to identify additional studies) in the search and date last searched.	3
Search	8	Present full search strategies for all electronic databases and other sources searched, including any limits used, such that they could be repeated.	3, Table S2
Study selection	9	State the process for selecting studies (i.e., screening, eligibility, included in systematic review, and, if applicable, included in the meta-analysis).	4
Data collection process	10	Describe method of data extraction from reports (e.g., piloted forms, independently, in duplicate) and any processes for obtaining and confirming data from investigators.	3,4
Definitions for data extraction	11	Provide definitions used in data extraction and classifications of target condition(s), index test(s), reference standard(s) and other characteristics (e.g. study design, clinical setting).	3,4
Risk of bias and applicability	12	Describe methods used for assessing risk of bias in individual studies and concerns regarding the applicability to the review question.	4
Diagnostic accuracy measures	13	State the principal diagnostic accuracy measure(s) reported (e.g. sensitivity, specificity) and state the unit of assessment (e.g. per-patient, per-lesion).	Table 1
Synthesis of results	14	Describe methods of handling data, combining results of studies and describing variability between studies. This could include, but is not limited to: a) handling of multiple definitions of target condition. b) handling of multiple thresholds of test	4

		positivity, c) handling multiple index test readers, d) handling of indeterminate test results, e) grouping and comparing tests, f) handling of different reference standards	
Meta-analysis	D2	Report the statistical methods used for meta-analyses, if performed.	4
Additional analyses	16	Describe methods of additional analyses (e.g., sensitivity or subgroup analyses, meta-regression), if done, indicating which were pre-specified.	4
**RESULTS**			
Study selection	17	Provide numbers of studies screened, assessed for eligibility, included in the review (and included in meta-analysis, if applicable) with reasons for exclusions at each stage, ideally with a flow diagram.	4, Figure 1
Study characteristics	18	For each included study provide citations and present key characteristics including: a) participant characteristics (presentation, prior testing), b) clinical setting, c) study design, d) target condition definition, e) index test, f) reference standard, g) sample size, h) funding sources	5, Table 1
Risk of bias and applicability	19	Present evaluation of risk of bias and concerns regarding applicability for each study.	4, Figure 2
Results of individual studies	20	For each analysis in each study (e.g. unique combination of index test, reference standard, and positivity threshold) report 2x2 data (TP, FP, FN, TN) with estimates of diagnostic accuracy and confidence intervals, ideally with a forest or receiver operator characteristic (ROC) plot.	6, Figure 3, Figure 4, Figure S1, Figure S2
Synthesis of results	21	Describe test accuracy, including variability; if meta-analysis was done, include results and confidence intervals.	6
Additional analysis	23	Give results of additional analyses, if done (e.g., sensitivity or subgroup analyses, meta-regression; analysis of index test: failure rates, proportion of inconclusive results, adverse events).	6
**DISCUSSION**			
Summary of evidence	24	Summarize the main findings including the strength of evidence.	7
Limitations	25	Discuss limitations from included studies (e.g. risk of bias and concerns regarding applicability) and from the review process (e.g. incomplete retrieval of identified research).	7,8
Conclusions	26	Provide a general interpretation of the results in the context of other evidence. Discuss implications for future research and clinical practice (e.g. the intended use and clinical role of the index test).	7,8
**FUNDING**			
Funding	27	For the systematic review, describe the sources of funding and other support and the role of the funders.	Table S3

Table S2. Searching strategy to find relevant articles.

**Database: PubMed**<br><br>((deep Learning) OR (artificial intelligence) OR (machine learning) OR (computer Aided) or (neural networks) OR (image classification) OR (object detection) OR (semantic segmentation)) AND ((ultrasonography) OR (ultrasound) OR (EUS)) AND ((pancreas) OR (pancreatic) OR (pancreas))
**Database: Embase**<br><br>('artificial intelligence':ti,ab,kw OR 'deep learning':ti,ab,kw OR 'machine learning':ti,ab,kw OR 'computer aided':ti,ab,kw OR 'neural networks':ti,ab,kw OR 'image classification':ti,ab,kw OR 'semantic segmentation':ti,ab,kw OR 'object detection':ti,ab,kw) AND ('ultrasonography':ti,ab,kw OR 'ultrasound':ti,ab,kw OR 'EUS':ti,ab,kw) AND ('Pancreas':ti,ab,kw OR 'Pancreatic':ti,ab,kw OR 'Pancreas':ti,ab,kw)
**Database: Cochrane Library**<br><br>#1 (artificial intelligence):ti,ab,kw OR (deep learning):ti,ab,kw OR (machine learning):ti,ab,kw OR (computer Aided):ti,ab,kw OR (neural networks):ti,ab,kw<br>#2 (image classification):ti,ab,kw OR (semantic segmentation):ti,ab,kw OR (object detection):ti,ab,kw<br>#3 #1 OR #2<br>#4 (ultrasonography):ti,ab,kw OR (ultrasound):ti,ab,kw OR (EUS):ti,ab,kw<br>#5 (pancreas):ti,ab,kw OR (pancreatic):ti,ab,kw OR (pancreas):ti,ab,kw<br>#6 #3 AND #4 AND #5
**Database: Web of Science**<br><br>(TS=(Artificial Intelligence) OR TS=(Deep Learning) OR TS=(Machine Learning) OR TS=(Computer Aided) OR TS=(neural networks) OR TS=(image classification) OR TS=(object detection) OR TS=(semantic segmentation)) AND (TS=(ultrasonography) OR TS=(ultrasound) OR TS=(EUS)) AND (TS=(pancreas) OR TS=(pancreatic) OR TS=(pancreas))

Table S3. Additional information of the included studies.

Study	Aims	Participant characteristics	Lesion type	Funding sources
Tonozuka 2021 (26)	Differentiate between PDAC, CP, and normal pancreas.	Total number of patients 139<br>Number of PDAC/CP/NP 76/34/29<br>Age, years, median (range) 66.0 (33-91)<br>Male/Female 43/33	Solid lesions	This study was supported by technical assistance and funding from Olympus Medical Corporation.
Udriştoiu 2021 (27)	Differentiate between CPP, PNET, and PDAC.	Total number of patients 65<br>Number of PDAC/CPP/PNET 30/20/15	Solid lesions	This study was supported by funding from the Norwegian Ministry of Education.
Oh 2021 (28)	Automatic segmentation of pancreatic cystic lesions.	Dataset A<br>Number of patients 52<br>Male/Female 20/32<br>Images 57<br>Dataset B<br>Number of patients 59<br>Male/Female 36/23<br>Images 364	Cystic lesions	This research was funded by the Korean government.
Huang 2022 (29)	Predict the preoperative invasiveness of PNET.	Total number of patients 104<br>Age, years 48.0 ± 12<br>Male/Female 56/48	Solid lesions	None
Kuwahar a 2022 (30)	Differentiate between PDAC, PASC, ACC, MPT, NEC, NET, SPN, CP and AIP.	Training cohort<br>Number of patients 694<br>Age, years, median (range), 67(15-90)<br>Male/Female 423/271<br>Validation cohort<br>Number of patients 78<br>Age, years, median (range), 66(33-88)<br>Male/Female 53/25<br>Test cohort<br>Number of patients 161<br>Age, years, median (range), 69(28-87)<br>Male/Female 83/78	Solid lesions	This research was funded by the Japan Society for the Promotion of Science.
Tian 2022 (31)	Differentiate between PDAC, PACC, PNET, PPC, SCA, SPN and IPMN.	Total number of patients 157<br>PC (n=102)<br>Male/Female 64/38<br>Age, years 63.36 ± 0.87<br>Non-PC (n=55)<br>Male/Female 35/20<br>Age, years 57.47 ± 1.5	Solid and cystic lesions	This research was supported by four foundations, including the National Natural Science Foundation of China.
Tong 2022 (32)	Differentiate between PDAC and CP.	Training cohort (n=351)<br>PDAC (n=264)<br>Male/Female 161/103<br>Age, years, 64±9<br>CP (n=87)<br>Male/Female 45/42<br>Age, years, 62±10<br>Internal validation cohort(n=109)<br>PDAC (n=73)<br>Male/Female 39/34<br>Age, years, 63±11<br>CP (n=36)<br>Male/Female 21/15<br>Age, years, median, 63±10<br>External validation cohort1(n=50)<br>PDAC (n=39)<br>Male/Female 25/14<br>Age, years, median, 67±11	Solid lesions	Several foundations, including the National Natural Science Foundation of China, supported this research.

		CP (n=11)<br>Male/Female 6/5<br>Age, years, median, 66±10<br>External validation cohort 2(n=48)<br>PDAC (n=38)<br>Male/Female 26/12<br>Age, years, median, 59±12<br>CP (n=10)<br>Male/Female 5/5<br>Age, years, median, 48±14		
VilasBoas 2022 (33)	Differentiate between mucinous versus non-mucinous pancreatic cystic lesions.	Total number of patients 28<br>Mucinous PCL (n=17)<br>Male/Female 7/10<br>Age, years, median (range), 64(53-69.5)<br>Non-Mucinous PCL (n=11)<br>Male/Female 5/6<br>Age, years, median (range), 64(53-72)	Cystic lesions	The Portuguese Foundation for Science and Technology supported this research.
Seo 2022 (34)	Automatic segmentation of pancreatic cancer.	Total number of patients 150<br>Male/Female 96/54<br>Age, years, median (range), 71.2 (49-94)	Solid lesions	The Korea Medical Device Development Fund supported this research.
Tang 2023 (35)	Differentiate between pancreatic cancer and benign pancreatic masses.	Experimental group (n=16)<br>Age, years, median, 68.19±12.34<br>Male/Female 5/11<br>Control group (n=23)<br>Age, years, median, 59.30±12.34<br>Male/Female 8/15	Solid lesions	Several grants funded this research, including the Natural Science Foundation of Hunan Province.

ACC, acinar cell carcinoma; AIP, autoimmune pancreatitis; CP, chronic pancreatitis; CPP, chronic pseudotumoral pancreatitis; IPMN, intraductal papillary mucinous neoplasm; MPT, metastatic pancreatic tumor; NEC, neuroendocrine carcinoma; NET, neuroendocrine tumor; PACC, pancreatic acinar cell carcinoma; PASC, pancreatic adenosquamous carcinoma; PDAC, Pancreatic ductal adenocarcinoma; PNET, Pancreatic neuroendocrine; PPC, pancreatic pseudocyst; SCA, pancreatic serous cystadenoma; SPN, solid pseudopapillary neoplasm.
